# Mitochondrial dysfunction in fatty acid oxidation disorders: insights from human and animal studies

**DOI:** 10.1042/BSR20150240

**Published:** 2016-01-15

**Authors:** Moacir Wajner, Alexandre Umpierrez Amaral

**Affiliations:** * Departamento de Bioquímica, Instituto de Ciências Básicas da Saúde, Universidade Federal de Rio Grande do Sul, Porto Alegre, RS 90035-003, Brazil; † Serviço de Genética Médica, Hospital de Clínicas de Porto Alegre, Porto Alegre, RS 90035-903, Brazil

**Keywords:** calcium homoeostasis, energy metabolism, fatty acids, fatty acid oxidation disorders, mitochondrial dysfunction, redox homoeostasis

## Abstract

Mitochondrial fatty acid oxidation (FAO) plays a pivotal role in maintaining body energy homoeostasis mainly during catabolic states. Oxidation of fatty acids requires approximately 25 proteins. Inherited defects of FAO have been identified in the majority of these proteins and constitute an important group of inborn errors of metabolism. Affected patients usually present with severe hepatopathy, cardiomyopathy and skeletal myopathy, whereas some patients may suffer acute and/or progressive encephalopathy whose pathogenesis is poorly known. In recent years growing evidence has emerged indicating that energy deficiency/disruption of mitochondrial homoeostasis is involved in the pathophysiology of some fatty acid oxidation defects (FAOD), although the exact underlying mechanisms are not yet established. Characteristic fatty acids and carnitine derivatives are found at high concentrations in these patients and more markedly during episodes of metabolic decompensation that are associated with worsening of clinical symptoms. Therefore, it is conceivable that these compounds may be toxic. We will briefly summarize the current knowledge obtained from patients and genetic mouse models with these disorders indicating that disruption of mitochondrial energy, redox and calcium homoeostasis is involved in the pathophysiology of the tissue damage in the more common FAOD, including medium-chain acyl-CoA dehydrogenase (MCAD), long-chain 3-hydroxyacyl-CoA dehydrogenase (LCHAD) and very long-chain acyl-CoA dehydrogenase (VLCAD) deficiencies. We will also provide evidence that the fatty acids and derivatives that accumulate in these diseases disrupt mitochondrial homoeostasis. The elucidation of the toxic mechanisms of these compounds may offer new perspectives for potential novel adjuvant therapeutic strategies in selected disorders of this group.

## Abstract (precis)

Patients affected by FAOD commonly present with hepatopathy, cardiomyopathy, skeletal myopathy and encephalopathy. Human and animal evidences indicate that mitochondrial functions are disrupted by fatty acids and derivatives accumulating in these disorders, suggesting that lipotoxicity may contribute to their pathogenesis.

## INTRODUCTION

Fatty acid oxidation defects (FAOD) are inherited metabolic diseases caused by deficiency of specific enzyme activities or transport proteins involved in the mitochondrial catabolism of fatty acids, leading to tissue accumulation of characteristic fatty acids and L-carnitine derivatives [41]. The more common disorders of this group are medium-chain acyl-CoA dehydrogenase (MCAD), long-chain 3-hydroxylacyl-CoA dehydrogenase (LCHAD) and very long-chain acyl-CoA dehydrogenase (VLCAD) deficiencies. The clinical findings are highly variable, ranging from multi-organ failure in newborns associated with a high mortality rate to late onset milder phenotypes [1–6]. Affected individuals usually present with hepatopathy, cardiomyopathy and skeletal myopathy, since mitochondrial fatty acid β-oxidation (FAO) is very active in liver, heart and skeletal muscle. Acute toxic encephalopathy presenting with seizures, hypotonia, lethargy and coma, as well as progressive neurologic deterioration with loss of intellectual function also occur in some of these disorders. In general, acute symptoms appear during catabolic situations such as infections, fasting and prolonged exercise when energy from FAO is most needed and the concentrations of the accumulating metabolites substantially increase due to their mobilization from the adipose tissue. Hypoglycemia due to reduced gluconeogenesis and increased tissue glucose uptake is also a major finding in FAOD [7].

Diagnosis of FAOD is usually carried out by measuring characteristic fatty acids, as well as their carnitine and glycine derivatives that accumulate in blood and urine. The gold standard diagnosis is performed by blood acylcarnitine analysis using Tandem mass spectrometry, although increased concentrations of dicarboxylic acids and glycine conjugates in urine measured by gas chromatography coupled to mass spectrometry is also helpful especially during acute illness. Functional studies on FAO and enzyme activity determination carried out in lymphocytes and/or fibroblasts, as well as molecular analyses may also be needed to achieve a conclusive diagnosis.

Therapy requires dietary restriction of fatty acid substrates, frequent meals to prevent catabolism and in certain cases L-carnitine supplementation (secondary L-carnitine deficiency and in the carnitine transporter defect–OCTN2) in order to avoid accumulation of toxic metabolites and hypoglycemia. It is also crucial to stop catabolic crises precipitated by infections by promptly and vigorously treating patients during these episodes with adequate supply of calories especially from carbohydrates to support anabolism. These measures may lead to an excellent prognosis for some FAOD, particularly MCAD deficiency, although they are still insufficient for other disorders. The role of L-carnitine supplementation is still controversial since it may not normalize tissue concentrations of this compound and may induce the production of potentially toxic long-chain acylcarnitines [8–10]. Bezafibrate, that is able to improve mitochondrial functions, has been utilized in VLCAD deficiency. Since outcome is usually improved by early diagnosis and treatment, the more common FAOD, i.e., MCAD and VLCAD deficiencies, were included in the neonatal mass screening programmes, helping to reduce mortality and morbidity in many children [4,11].

The pathophysiology of FAOD is not yet fully established, although energy deficiency seems to play an important role especially in the hepatopathy and cardiomyopathy of the affected patients [8,12]. It is of note that central and peripheral neuropathy cannot be corrected by high caloric intake and progressive and acute neurologic symptoms are not always associated with hypoglycemia. Therefore, it is conceivable that other mechanisms than energy deprivation may be implicated in the pathogenesis of these disorders. In this scenario, hyperammonemia, depletion of free L-carnitine and/or CoA [13], oxidative stress and accumulation of toxic lipids (lipotoxicity) may also potentially compromise the normal functioning of various tissues in FAOD [8,12]. It is expected that the elucidation of the exact pathogenetic mechanisms will allow the development of novel therapeutic strategies to benefit the affected patients.

This short review will summarize the accumulating evidence indicating that mitochondrial dysfunction contributes to the pathophysiology of the more common FAOD, such as MCAD, LCHAD and VLCAD deficiencies. We will focus on the toxic properties of the accumulating fatty acids and carnitine derivatives disrupting mitochondrial functions.

## MEDIUM-CHAIN ACYL-CoA DEHYDROGENASE DEFICIENCY

MCAD (EC 1.3.99.3) deficiency (OMIM # 201450), the most common FAOD with a prevalence of 1:10,000 to 1:27,000 newborns, is caused by deficient activity of the flavoenzyme MCAD. It is biochemically characterized by accumulation of high amounts of octanoate, decanoate, *cis*-4-decenoate and their carnitine derivatives, as well as by lactic acidosis during episodes of metabolic decompensation [14].

Affected children are normal at birth, but usually develop severe symptoms that may lead to a fatal outcome in 20–40% of the cases in the first 5 years of age [15]. Clinical presentation usually occurs during fasting or other situations involving metabolic stress and are characterized by lethargy, seizures and coma as well as by hypoketotic hypoglycemia. Hepatomegaly and acute liver disease with hyperammonemia may also appear during crises. Progressive encephalopathy with brain abnormalities is found in many untreated patients [16]. Sometimes this disorder is misdiagnosed as Reye syndrome because of their similar neurological manifestations and the accumulation of octanoic acid in both pathologies [17–21]. Late onset presentations also occur at any age, even in adulthood [22,23]. The prognosis is excellent once the diagnosis is established by neonatal screening. Low availability of brain substrates (glucose and ketones) combined with hyperammonemia and the potentially toxic accumulating medium-chain fatty acids and/or derivatives were hypothesized to lead to disruption of brain energy functions and the development of encephalopathy [1,24]. Chronic muscle weakness and rhabdomyolisis during acute episodes can be also observed [25,26].

Diagnosis should consider the clinical status of the patients (acutely symptomatic or asymptomatic). It is usually performed by the detection of increased octanoylcarnitine in blood, as well as urinary medium-chain dicarboxylic acids and glycine derivatives (hexanoylglycine, suberylglycine and phenylpropionylglycine in urine [14].

The major therapy goal is to reverse catabolism and sustain anabolism by giving simple carbohydrates by mouth or intravenously. Avoidance of fasting is critical to prevent clinical manifestations, so that infants require frequent feedings. L-Carnitine sometimes coupled to riboflavin supplementation may be helpful in MCAD deficiency, although this is still controversial. L-Carnitine was shown to reduce the number and severity of metabolic decompensation in some patients by correcting the secondary deficiency of this compound and probably by its property of binding to the toxic accumulating metabolites increasing their urinary excretion. L-Carnitine may also restore acyl-CoA/CoA ratio that is necessary for crucial mitochondrial functions [27–29]. On the other hand, riboflavin was shown to activate octanoyl-CoA dehydrogenase in lymphocytes from MCAD-deficient patients [28,30]. However, it is important to note that clinical improvement by these supplements is still unproven [9], although some reports demonstrate that riboflavin and L-carnitine improves the biochemical phenotype of MCAD-deficient patients.

## LONG-CHAIN 3-HYDROXYACYL-CoA DEHYDROGENASE DEFICIENCY

LCHAD (EC 1.1.1.211) deficiency (OMIM # 609016) has a heterogeneous clinical presentation, varying from sudden infant death to milder cases or even a benign course [31]. It was first described in 1989 [32] and has an approximate incidence of 1:50,000 newborns [33]. Common features of the severe form of this disorder include hypoglycemia, metabolic acidosis, hyperlactic acidemia, hyperammonemia, skeletal myopathy, hypotonia, cardiomyopathy and hepatopathy, as well as fat tissue accumulation. Mortality mainly caused by cardiac decompensation and liver failure may be as high as 80% in the first years of life [4]. Milder cases surviving into adolescence and adulthood usually present hypotonia, seizures, mental retardation, hypoglycemia, cardiomyopathy, peripheral neuropathy and retinopathy [4,34]. Metabolic crises are characterized by encephalopathy with seizures, hypoketotic hypoglycemia, vomiting and dehydration precipitated by infections. Diagnosis by newborn screening (NBS) followed by early treatment may not prevent symptomatology in many children [35]. In contrast, NBS was demonstrated to significantly reduce morbidity and mortality in patients with mitochondrial trifunctional protein (MTP) [36], a clinically and biochemically disorder undistinguishable from LCHAD deficiency.

Diagnosis of this disease is based on the identification of high urinary excretion of dicarboxylic acids with a hydroxy group and their carnitine derivatives in blood, as well as on functional studies of long-chain fatty acids and measurement of the enzyme activity in fibroblasts [14].

Therapy includes prevention of fasting and acute infections, as well as a high carbohydrate and low fat consumption at frequent intervals combined with medium-chain triacylglycerols (MCT) supplementation [37]. The administration of L-carnitine does not prevent the fatal course of cardiac decompensation and could even aggravate the clinical condition possibly by generating toxic long-chain carnitine derivatives [38,39]. This is in line with the findings that long-chain acylcarnitines were shown to provoke arrhythmogenic effects [40], so that L-carnitine utilization as an adjuvant therapy should be cautiously evaluated and debated.

## VERY LONG-CHAIN ACYL-CoA DEHYDROGENASE DEFICIENCY

VLCAD (EC 1.3.99.3) deficiency (OMIM # 609575) is considered the most common defect of the mitochondrial oxidation of long-chain fatty acids with an incidence of 1:30,000 to 1:100,000 [41–45]. Patients present with heterogeneous clinical phenotypes affecting mainly heart, liver and skeletal muscle. Common findings are hepatomegaly, cardiomyopathy and hypoketotic hypoglycemia that are commonly induced by prolonged fasting and infectious illnesses [46–48]. Skeletal myopathy associated with rhabdomyolysis may also be precipitated by vigorous exercise [49,50]. Initially, this disorder was diagnosed as LCAD deficiency, but most affected patients were shown later to be VLCAD deficient [51].

Diagnosis is based on urine organic acid analysis, which reveals increased amounts of saturated and unsaturated dicarboxylic acids. Furthermore, blood acylcarnitine profile analysis is also important showing elevated 5-*cis*-tetradecenoylcarnitine (C14:1). Functional studies in lymphocytes/fibroblasts sometimes combined with molecular analyses are also required for diagnostic confirmation [14].

The recommended therapeutic approach includes the replacement of long-chain triacylglycerols by MCT, which can be fully oxidized in the mitochondrial β-oxidation pathway. The clinical efficacy of MCT is widely recognized especially with respect to the prevention and treatment of cardiomyopathy and muscular symptoms [37,50]. Although MCT diet is considered a safe dietary intervention and is applied in various FAOD for longer periods, recent reports highlight the adverse effects of this diet in the murine model of VLCAD deficiency [52–54]. Long-term supplementation over one year contributed to the development of an unexpected clinical phenotype with an increased body fat content and a disturbance in body fat composition [52].

On the other hand, supplementation of odd-chain triacylglycerols [50] and bezafibrate, a stimulator of mitochondrial functions, was shown to improve skeletal myopathy and rhabdomyolysis [55], although this is still disputed [56,57].

The inclusion of this disorder in the neonatal screening programmes helped to prevent the life-threatening symptoms associated with hypoglycemia and cardiomyopathy in a significant number of patients, whereas others remained asymptomatic [35,37].

## FAOD PATHOGENESIS

Although various mechanisms have been proposed to explain liver, heart, skeletal muscle and brain dysfunction in FAOD, the pathogenesis of these disorders is not fully established. Inadequate energy supply due to a blockage of FAO combined with hypoketotic hypoglycaemia seems to be central in the pathophysiology of tissue damage and more particularly in the cardiomyopathy and skeletal myopathy in patients affected by FAOD [8,12]. Energy deprivation is probably accentuated by sequestration of Co A and L-carnitine. However, since energetic substrate supplementation is not able to reverse or prevent symptomatology in some patients, it is presumed that other pathogenetic mechanisms are implicated. Thus, it has been postulated that misfolded proteins leading to oxidative stress and loss of protein–protein interaction that are observed in short-chain acyl-CoA dehydrogenase (SCAD), SCHAD and MCAD-deficient patients, as well as the toxic effects of high ammonia levels may also be implicated in FAOD pathophysiology [12,58]. More recently, growing evidence is emerging pointing to the toxicity of the accumulating fatty acids and derivatives. In the present review, we stress the role of disruption of mitochondrial homoeostasis revelled by deficient energy production and oxidative stress in humans and genetic animal models of FAOD. We also provide experimental data demonstrating that disruption of mitochondrial functions is caused by fatty acids and acylcarnitines found at high concentrations in tissues of the affected patients. This is consistent with the observations showing that catabolic events that are characterized by substantial increase in the potentially toxic fatty acids and derivatives are usually associated with worsening of the myocardiopathy, skeletal myopathy and hepatopathy.

## DISRUPTION OF MITOCHONDRIAL HOMOEOSTASIS IN FAOD

Mitochondria are crucial organelles for cellular homoeostasis and survival, participating in energy (ATP) production and intracellular transfer, as well as in the regulation of redox and calcium homoeostasis, FAO and apoptosis [59,60]. Oxidative phosphorylation (OXPHOS) is the major source of cellular ATP and reactive oxygen species (ROS) formed during electron flow through the respiratory chain [61]. ROS have essential functions in cellular signalling mainly by regulating the expression/activity of many genes and enzymes. However, when at high concentrations, ROS become toxic to the cell causing oxidative damage to mitochondrial proteins, lipids and DNA that may lead to a cascade of apoptosis or necrosis [62]. Oxidative damage and elevated intracellular calcium concentrations cause mitochondrial stress and collapse of internal membrane potential in a process called mitochondrial permeability transition (mPT) that also leads to cell death [63].

Heart, liver, skeletal muscle and brain are highly dependent on OXPHOS for energy production and therefore are highly susceptible to alterations of mitochondrial function. When OXPHOS is compromised, ATP synthesis is decreased and free radical production increased, potentially leading to cell damage. Thus, it is expected that disordered mitochondrial functions is associated with cardiomyopathy, hepatopathy, skeletal myopathy and encephalopathy.

Primary disorders of mitochondrial functions can be caused by mutations in either mitochondrial DNA or nuclear genes, whereas secondary mitochondrial alterations are due to endogenous or exogenous toxins disrupting mitochondrial homoeostasis. In this context, over the last decades there has been an increasing recognition that mitochondrial dysfunction plays an important role in the pathophysiology of human diseases and more recently in FAOD [64–66].

In this review, we will summarize the available data of the literature from humans and animals (genetic mouse models) with MCAD, LCHAD and VLCAD deficiencies, supporting the presumption that mitochondrial dysfunction represents a relevant contributing mechanism of the pathogenesis of these disorders. We will also give solid evidence on the toxicity of fatty acids and carnitine derivatives that accumulate in these disorders disrupting mitochondrial homoeostasis.

## HUMAN EVIDENCE THAT MITOCHONDRIAL DYSFUNCTION IS INVOLVED IN FAOD PATHOGENESIS

Table 1 displays biochemical and morphological alterations observed in tissues of patients affected by the more common FAOD, i.e., MCAD, LCHAD and VLCAD deficiencies, strongly suggesting that mitochondrial dysfunction contributes to the pathogenesis of these disorders. Intermittent or persistent elevations of lactic acid in plasma, decreased activity of respiratory chain complexes, oxidative stress biomarkers, mitochondrial morphological abnormalities and rhabdomyolysis visualized on light or electron microscopy are shown in the table [26,28,29,38,49,50,67–77].

**Table 1:** Human evidence that mitochondrial dysfunction is involved in the pathophysiology of MCAD, LCHAD and VLCAD deficiencies

**MCAD deficiency**	
Induction of oxidative stress	[28,77]
Hyperlactic acidemia	[72,74]
Rhabdomyolysis	[26]
**LCHAD deficiency**	
Induction of oxidative stress	[75]
Mitochondrial abnormalities	[71]
Respiratory chain inhibition	[67,71,73]
Hyperlactic acidemia	[67,71–73]
Rhabdomyolysis	[3,76]
**VLCAD deficiency**	
Hyperlactic acidemia	[72]
Rhabdomyolysis	[49,50,76]

## ANIMAL EVIDENCE OF MITOCHONDRIAL DYSFUNCTION IN FAOD

Animal models of FAOD were developed to gain insights into the mechanisms of pathogenesis of these diseases in order to allow the development of better therapy for human patients.

Table 2 shows the general characteristics of the available FAOD animal models [78–89], whereas Table 3 displays mitochondrial alterations observed in some of these models. The genetic mouse model of VLCAD deficiency (VLCAD^−/−^) is the most utilized model investigating the pathogenesis of this disease. In contrast, the high neonatal mortality of the animal model of MCAD deficiency makes the study of long-term pathogenesis in this model rather difficult [87]. MTP-deficient mice also have a high mortality in the first 36 h of life probably due to cardiorespiratory insufficiency, making this model inappropriate to study long-term pathogenesis of this disorder [82,90].

**Table 2:** Genetic knockout mouse models of FAOD

Enzyme deficiency	Biochemical and histopathological phenotypes	References
SCAD	Increase in ethylmalonic and methylsuccinic acids and *N*-butyrylglycine in urine Fatty liver disease	[78]
MCAD	Increase in hexanoyl carnitine, octanoylcarnitine, decanoylcarnitine and *cis*-4-decenoylcarnitine in plasma Neonatal mortality	[87]
LCAD	Increase in free fatty acids and carnitine derivatives of C12:1, C14:1, C14:2, C18:1, C18:2, hyperlactic acidemia and hypoglycemia Cardiac and hepatic alterations	[79] [80]
VLCAD	Low concentrations of free carnitine in blood and accumulation of long-chain acylcarnitines in tissues Increase in free fatty acids in blood Cardiac, hepatic and muscular alterations	[81,89] [83] [84,85]
MTP α subunit LCHAD	Increase in free fatty acids and carnitine derivatives of C14, C14:1, C16, C16:1, C18:1, C18:2 and hypoglycemia Cardiac and hepatic alterations	[82]
CPT–1a liver	Homozygous are not viable	[86]
CPT–1b muscle	Homozygous are not viable	[88]

**Table 3:** Evidence of mitochondrial dysfunction in genetic mouse models of FAOD

Enzyme deficiency	Mitochondrial dysfunction	References
SCAD	Mitochondrial swelling and microvesicular fatty changes in hepatocytes Respiratory chain complex alterations	[92] [94]
LCAD	Low concentrations of citric acid cycle intermediates	[93]
VLCAD	Abnormal mitochondrial bioenergetics (uncoupled mitochondria, increase in glucose uptake and decrease in phosphocreatine/ATP ratio) Induction of oxidative stress	[54,91] [52]
MTP α subunit LCHAD	Swelling and distortion of mitochondria Induction of oxidative stress	[82] [90]

Although these models revealed multiple mechanisms involved in the pathophysiology of FAOD [8,54], mitochondrial alterations and disruption of redox homoeostasis were more commonly observed [8,54,82,91–94].

It is of note that LCAD and VLCAD metabolize long-chain fatty acids in the mice, whereas VLCAD is the active enzyme in humans, so that there are only patients affected by deficient activity of VLCAD. Moreover, although deficiencies of these enzymes in the mice have some similarities to VLCAD deficiency in humans, they are not identical. It is stressed that the LCAD genetic mouse model (LCAD^−/−^) has a more severe phenotype than the VLCAD^−/−^ mice. LCAD^−/−^ mice accumulate the same acylcarnitines as those of VLCAD^−/−^ patients on food withdrawal and may have cardiac hypertrophy at birth and hypoketotic hypoglycemia with marked fatty acid deposition in liver and heart [81]. Furthermore, sudden death occurs in some LCAD^−/−^ mice during conditions of no apparent external stress [80]. Regarding to the VLCAD^−/−^ mice, there is no characteristic clinical phenotype at rest but when these animals are submitted to vigorous exercise, fasting or ice-cold exposure they have similar stress-induced phenotypes as humans, including severe hypoglycemia, hypothermia and lethargy, as well as to a fatal outcome in one third of them [81,85]. Disruption of mitochondrial bioenergetics with liver and heart steatosis and bradycardia has been also observed in VLCAD^−/−^ mice submitted to fasting, ice-cold and severe hypoglycemia [91]. These animals also develop progressive cardiac dysfunction due to chronic energy deficiency evidenced by reduction in phosphocreatine/ATP ratio [54].

On the other hand, it has been demonstrated that energy supply given by high dietary MCT intake fails to improve and even aggravates cardiac performance inducing dilated cardiomyopathy in VLCAD deficiency, implying that other pathogenetic mechanisms than energy deficiency may underlie cardiac dysfunction in these patients [50]. Furthermore, MCT supplementation was a shown to induce oxidative stress in VLCAD-deficient animals [54]. This is consistent with the observations that fasting-induced hepatopathy in VLCAD-deficient mice was associated with ROS generation and up-regulation of peroxisomal and microsomal oxidation pathways that generate ROS and lipid peroxides potentially toxic to tissues [52,95].

Overall, the available human and animal studies point to mitochondrial dysfunction as one important mechanism in the pathogenesis of tissue damage in patients affected by FAOD. However, the underlying mechanisms of mitochondrial deregulation are still unclear in these disorders. We present below evidence that lipotoxicity caused especially by the major fatty acids, as well as by acylcarnitines accumulating in some FAOD may contribute decisively to disrupt mitochondrial homoeostasis.

## TOXICITY OF THE MAJOR METABOLITES ACCUMULATING IN FAOD

Considering that long-chain fatty acids normally present in plasma of normal individuals have cytotoxic effects when at high concentrations [96–101], it is feasible that the fatty acids and acylcarnitines found at high tissue concentrations in FAOD may behave similarly and induce cellular toxicity. This presumption is supported by mounting evidence of deleterious effects on mitochondrial functions attributed to these compounds. We will concentrate in this review on the toxicity of the accumulating metabolites in the more common FAOD, namely MCAD, LCHAD and VLCAD deficiencies.

Table 4 shows that medium-chain fatty acids accumulating in MCAD deficiency deregulate various crucial mitochondrial functions in brain, liver and skeletal muscle. It can be observed in the table that the medium-chain fatty acids inhibit energy production, utilization and transfer [102–106], uncouple OXPHOS [107–109] and induce oxidative stress [106,110,111], which may result at least partly from the blockage of the respiratory chain stimulating superoxide and other ROS production. The deleterious effects were more pronounced with decanoic acid and *cis*-4-decenoic acid, that also induced mPT, a condition that compromise all mitochondrial functions, including energy production, maintenance of cellular redox status and Ca^2+^ retention capacity, culminating in cell death [112]. In contrast, the medium-chain carnitine derivatives did not significantly impair mitochondrial homoeostasis, implying that they are less toxic to mitochondria as compared with their fatty acid analogues. In contrast, these carnitine derivatives were shown to induce oxidative stress in brain [113]. Therefore, it could be presumed that mitochondrial dysfunction provoked by the accumulating medium-chain fatty acids may contribute to the neurologic, muscular and hepatic symptoms found in MCAD-deficient patients.

**Table 4:** Toxicity of medium-chain fatty acids and carnitine derivatives on mitochondrial functions

Accumulating metabolites	Tissue	Mitochondrial homoeostasis disruption	References
Octanoic acid, decanoic acid	Brain	Uncoupling of OXPHOS Metabolic inhibition ↓ NAD(P)H content ↓ Respiratory chain activity ↓ Na^+^,K^+^–ATPase activity Induction of oxidative stress	[107,108] [108] [108] [103,105] [102] [110]
	Liver	↓ Respiratory chain activity Induction of oxidative stress Induction of permeability transition Reduction in Ca^2+^ retention capacity	[106] (A. U. Amaral, J. C. da Silva, A. Wajner, K. dos Santos Godoy, C. Cecatto and M. Wajner, Unpublished results)
	Skeletal muscle	↓ Respiratory chain activity Induction of oxidative stress	[106]
*cis*-4-Decenoic acid	Brain	Uncoupling of OXPHOS Metabolic inhibition ↓ NAD(P)H content ↓ Respiratory chain complex and creatine kinase activities ↓ Na^+^,K^+^–ATPase activity Induction of oxidative stress	[109] [109] [109] [105] [104] [111]
	Liver	Induction of permeability transition Reduction in Ca^2+^ retention capacity	(A. U. Amaral, J. C. da Silva, A. Wajner, K. dos Santos Godoy, C. Cecatto and M. Wajner, Unpublished results)
Octanoylcarnitine, decanoylcarnitine	Brain	Induction of oxidative stress	[113]
	Liver	Normal mitochondrial bioenergetics	(A. U. Amaral, J. C. da Silva, A. Wajner, K. dos Santos Godoy, C. Cecatto and M. Wajner, Unpublished results)

Although the exact pathogenesis of LCHAD deficiency is still obscure, a mitochondrial role is suggested based on the findings of decreased activities of single or multiple respiratory chain complexes that may possibly explain the hyperlactic acidemia observed in the patients. Table 5 shows that the major hydroxylated fatty acids accumulating in LCHAD deficiency disturb energy and redox homoeostasis in various animal tissues. These compounds were shown to uncouple OXPHOS and induce mPT pore opening, leading to deregulation of important mitochondrial functions such as maintenance of membrane potential, NAD(P)H redox status and calcium retention capacity in forebrain of adolescent rats [114,115], as well as to induce oxidative stress [116]. Similar but more intense effects were obtained in rat liver [117] and heart [118,119] mitochondria. These data allied to previous observations demonstrating that long-chain 3-hydroxyacyl-CoA derivatives inhibit ATP production in human fibroblasts [120] and to the evidence showing bioenergetics dysfunction in skeletal muscle of MTP-deficient patients [71], support the hypothesis that long-chain 3-hydroxy fatty acids and derivatives disrupt energy and redox mitochondrial homoeostasis, probably representing a relevant underlying mechanism in the pathophysiology of the cardiac, hepatic, myopathic and cerebral alterations observed in LCHAD deficiency.

**Table 5:** Toxicity of long-chain hydroxy fatty acids on mitochondrial functions

Accumulating metabolites	Tissue	Mitochondrial homoeostasis disruption	References
3-Hydroxydodecanoic acid	Brain	Weak uncoupling of OXPHOS Induction of oxidative stress	[114] [116]
3-Hydroxytetradecanoic acid	Brain	Uncoupling of OXPHOS Induction of permeability transition Induction of oxidative stress	[114] [115] [116]
	Liver	Uncoupling of OXPHOS	[117]
	Heart	Induction of permeability transition Reduction in Ca^2+^ retention capacity	[118] [119]
	Skeletal muscle	Induction of permeability transition	(C. Cecatto, K. dos Santos Godoy, J. C. da Silva, A. U. Amaral and M. Wajner, Unpublished results)
3-Hydroxypalmitic acid	Brain	Similar but more intense effects as compared with 3-hydroxytetradecanoic acid	[114–119]
	Liver		
	Heart		
	Skeletal muscle		(C. Cecatto, K. dos Santos Godoy, J. C. da Silva, A. U. Amaral and M. Wajner, Unpublished results)
3-Hydroxytetradecanodioic acid	Brain	No alterations	[118] [117] [119]
	Liver		
	Heart		

Table 6 displays the experimental animal evidence that long-chain fatty acids and carnitine derivatives accumulating in VLCAD deficiency deregulate various crucial mitochondrial functions in the heart. In this context, it was demonstrated that the carnitine derivatives uncouple OXPHOS [121] and disturb cellular calcium homoeostasis [122,123]. Furthermore, monounsaturated long-chain fatty acids accumulating in VLCAD deficiency were shown to decrease mitochondrial membrane potential and induce apoptosis and necrosis in cultured cardiomyocytes, supporting the hypothesis that these compounds are involved in the pathogenesis of the cardiac symptoms in this disease and contribute to the irreversible cardiac damage [124].

**Table 6:** Toxicity of long-chain fatty acids and carnitine derivatives accumulating in VLCAD deficiency on mitochondrial functions

Accumulating metabolites	Mitochondrial homoeostasis disruption		References
Long-chain acylcarnitines	Heart	Uncoupling of OXPHOS Increase in intracellular Ca^2+^ concentration	[121] [122,123]
Long-chain fatty acids	Heart	Decreased mitochondrial membrane potential Induction of apoptosis and necrosis	[124]

Taken together, the available data strongly indicate that some fatty acids and acylcarnitines accumulating in FAOD play an important role in the symptomatology and pathogenesis of affected patients. Therefore, it is conceivable that these compounds disrupt mitochondrial homoeostasis, especially during catabolic situations in which their concentrations significantly increase in blood and other tissues due to accelerated lipolysis.

**Figure 1:**
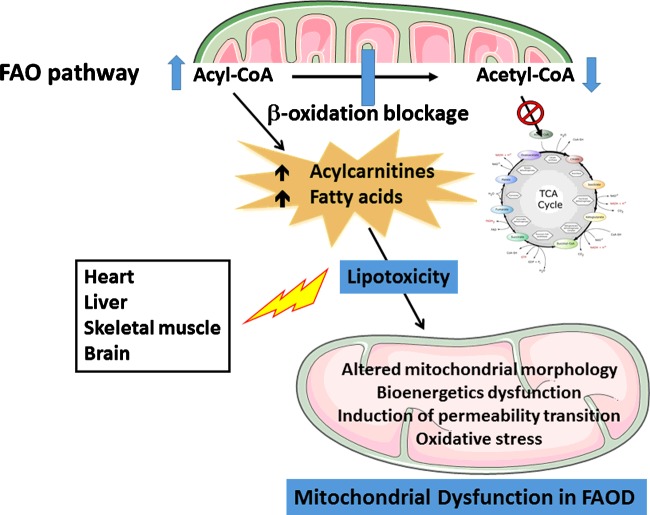
Mitochondrial dysfunction provoked by fatty acids and acylcarnitines accumulating in FAOD

Figure 1 depicts the potential mechanisms involved in FAOD pathophysiology, emphasizing the important role of lipotoxicity provoked by the accumulating metabolites inducing deregulation of mitochondrial homoeostasis.

## CONCLUDING REMARKS

Growing evidence obtained from human and animal studies revealed that disturbance of mitochondrial functions associated with oxidative stress are involved in the pathophysiology of FAOD. It is emphasized the toxic role of some fatty acids and acylcarnitine derivatives that accumulate in these disorders disrupting mitochondrial homoeostasis and therefore contributing to the chronic and the acute symptomatology seen in some of these defects. Further *in vitro* and particularly *in vivo* studies in animal models and humans are however necessary to substantiate this hypothesis. Moreover, although it is difficult to evaluate the relative contribution of the toxic fatty acids and derivatives in the pathology of these diseases, it is conceivable that there is a synergistic action between the toxicity of these metabolites, hyperammonemia, energy deficit and sequestration of CoA, finally leading to tissue damage. It is therefore expected that the development of new drugs targeting the mitochondrion, initially in animal models and thereafter as adjuvant therapeutic approaches for the patients, may become an important focus in the future. In this context, the antioxidant and anti-inflammatory natural compound resveratrol with beneficial properties on mitochondrial energy metabolism [125] and FAO [126] was shown to improve mitochondrial FAO capacities in fibroblasts from human VLCAD- and carnitine palmitoyltransferase (CPT)2-deficient patients by increasing the expression of VLCAD and CPT2 proteins. Thus, resveratrol may be a potential novel candidate for the treatment of these diseases by a dual mechanism, improving FAO and counteracting oxidative stress [127]. Other therapies such as bezafibrate and CoQ10 may also act synergistically with resveratrol helping to improve mitochondrial functions in FAOD.

## Abbreviations

FAO: fatty acid oxidation
FAOD: fatty acid oxidation defects
MCAD: medium-chain acyl-CoA dehydrogenase
LCHAD: long-chain 3-hydroxyacyl-CoA dehydrogenase
MCT: medium-chain triacylglycerols
mPT: mitochondrial permeability transition
MTP: mitochondrial trifunctional protein
NBS: newborn screening
OXPHOS: oxidative phosphorylation
ROS: reactive oxygen species
VLCAD: very long-chain acyl-CoA dehydrogenase
